# RETRACTION: A Novel Prognostic Pyroptosis-Related Gene Signature Correlates to Oxidative Stress and Immune-Related Features in Gliomas

**DOI:** 10.1155/omcl/9872327

**Published:** 2025-10-06

**Authors:** Oxidative Medicine and Cellular Longevity

RETRACTION: S. Zeng, W. Li, H. Ouyang, Y. Xie, X. Feng, and L. Huang, “A Novel Prognostic Pyroptosis-Related Gene Signature Correlates to Oxidative Stress and Immune-Related Features in Gliomas,” *Oxidative Medicine and Cellular Longevity* 2023 (2023): 4256116, https://doi.org/10.1155/2023/4256116.

The above article, published online on 31 January 2023 in Wiley Online Library (wileyonlinelibrary.com), has been retracted by agreement between the authors; journal Editor, Amjad Islam Aqib and John Wiley & Sons Ltd.

The retraction was requested by the authors due concerns around the validity of the data such that they no longer have confidence in the results or conclusions. Additionally, author Li Huang informed the journal that they did not contribute to this study.

